# Supplementary material for: Concurrent Activation of Both Survival-Promoting and Death-Inducing Signaling by Chloroquine in Glioblastoma Stem Cells: Implications for Potential Risks and Benefits of Using Chloroquine as Radiosensitizer
Source: Cells. 2023 Apr 30;12(9):1290. doi: 10.3390/cells12091290 (PMC10177603; doi:10.3390/cells12091290)
Supplement: Supplementary file 1 [file cells-12-01290-s001.zip › cells-2336826-supplementary.pdf]

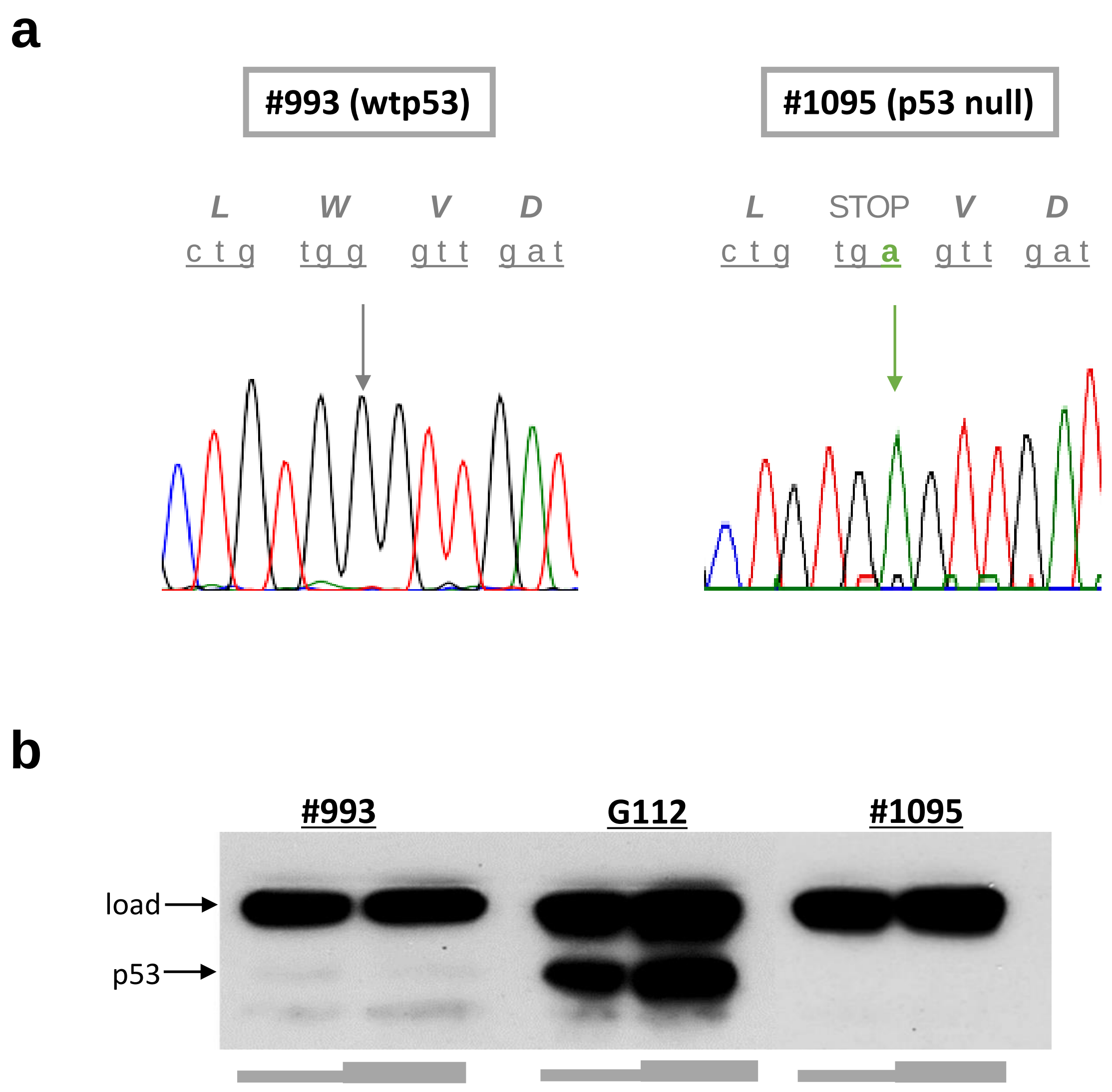
a
#993 (wtp53)
#1095 (p53 null)
L W V D
c t g tg g g t t g a t
L STOP V D
c t g tg a g t t g a t
b
#993
G112
#1095
load
p53

Figure S1

c

#993 (wtp53)

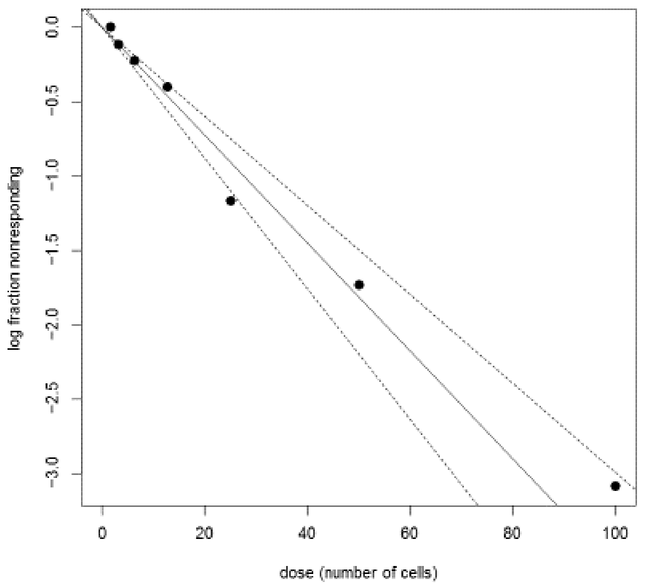

G112 (R273H-p53)

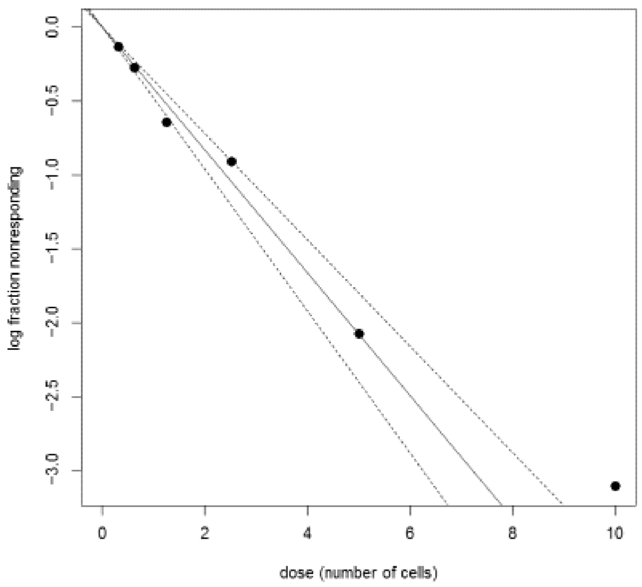

#1095 (p53 null)

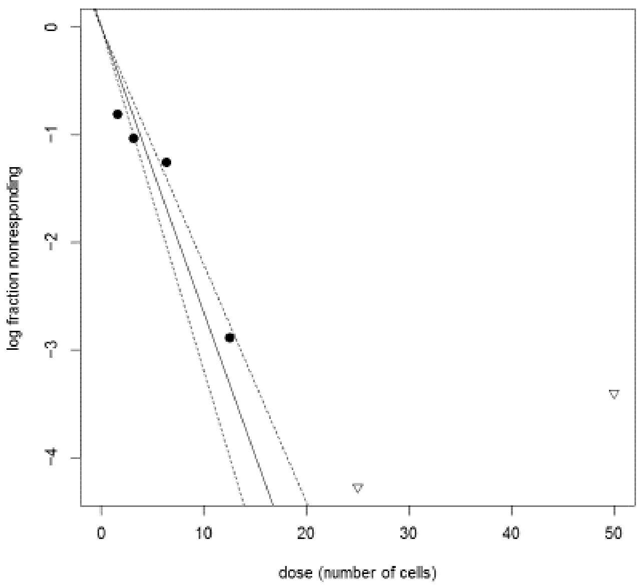

Group	Lower	Estimate	Upper
#993	33.4	27.6	22.8

Group	Lower	Estimate	Upper
G112SP	2.78	2.41	2.08

Group	Lower	Estimate	Upper
#1095	4.53	3.77	3.13

Figure S1

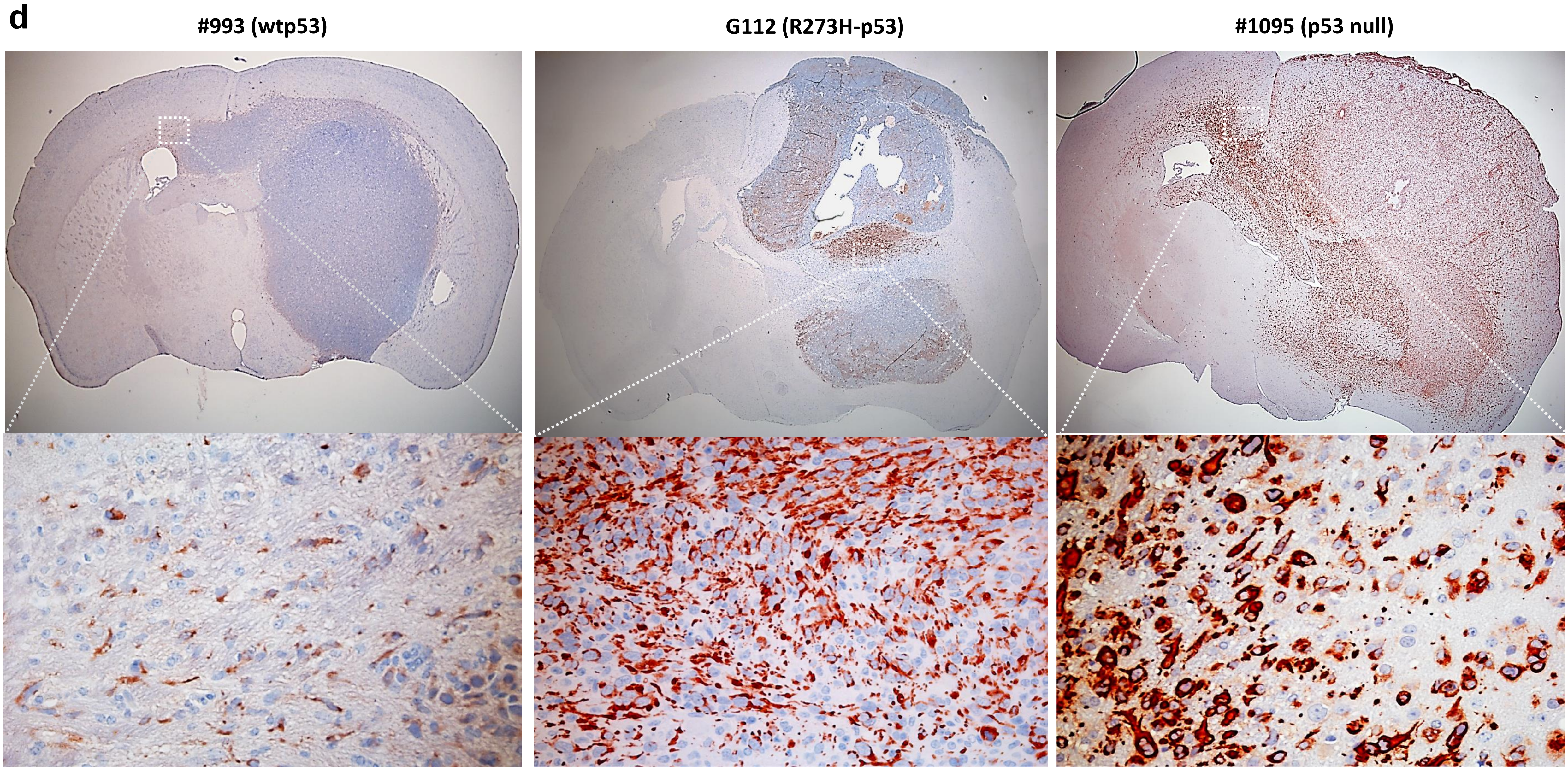

Figure S1

a)

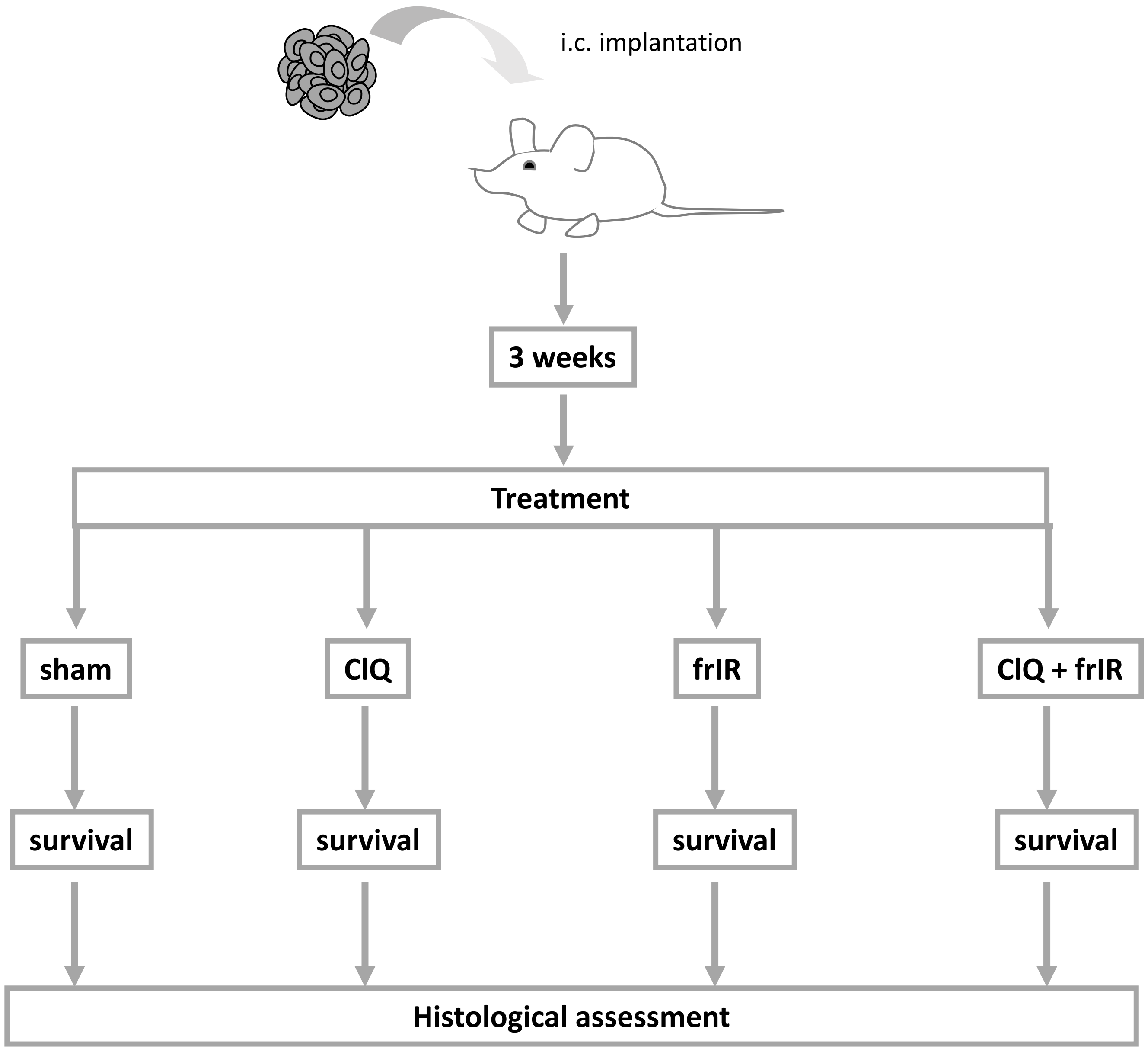

i.c. implantation
3 weeks
Treatment
sham
ClQ
frIR
ClQ + frIR
survival
survival
survival
survival
Histological assessment

Figure S2

b)

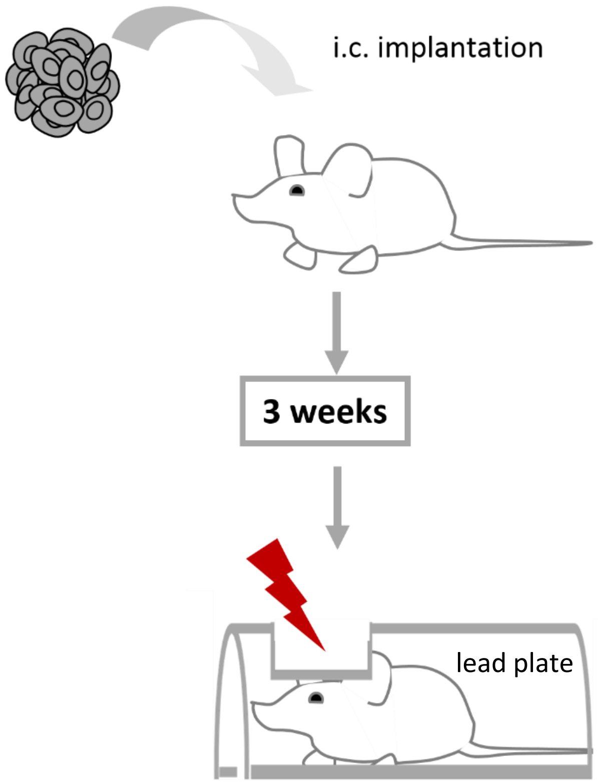

Figure S2

a

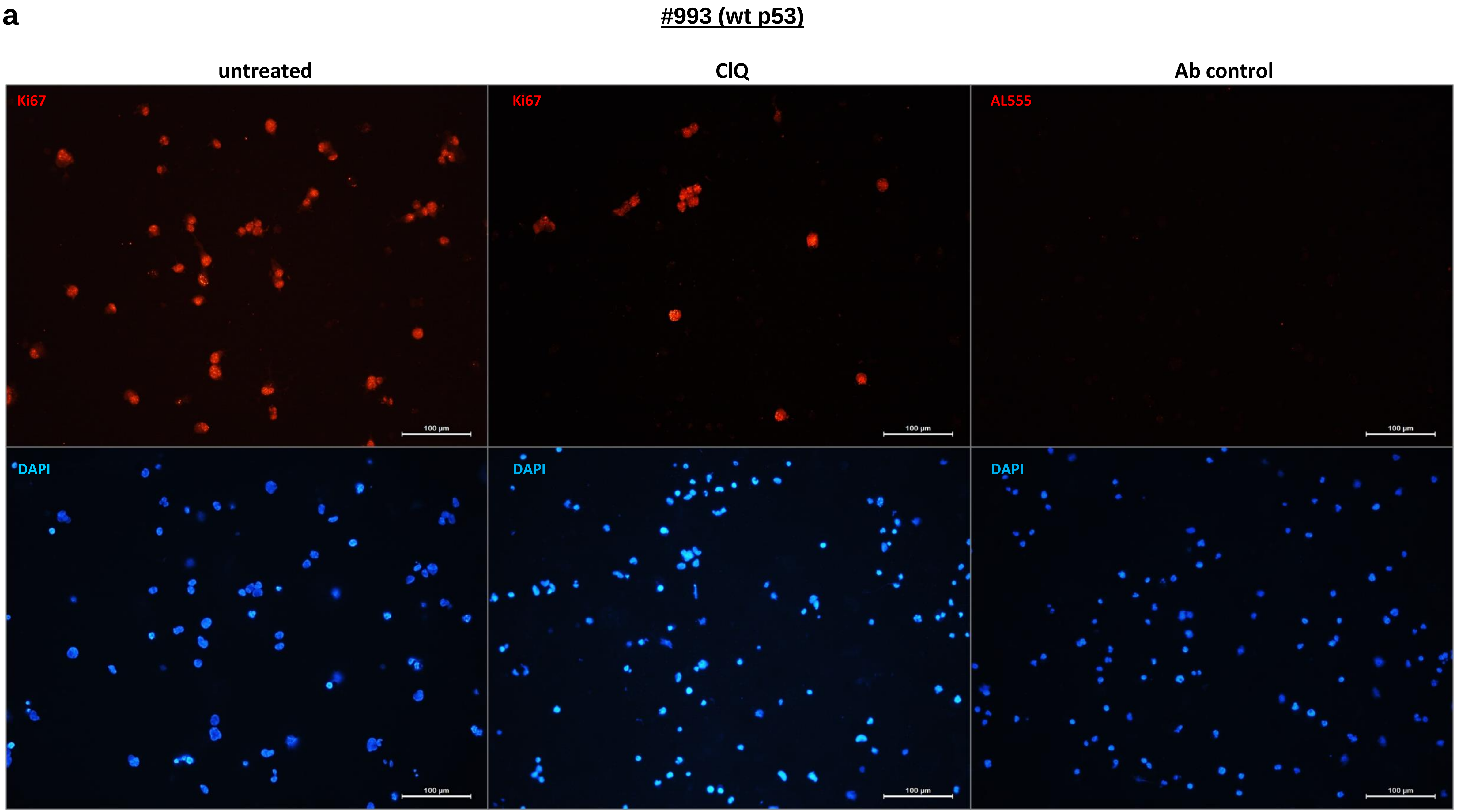

#993 (wt p53)
untreated
ClQ
Ab control
Ki67
Ki67
AL555
DAPI
DAPI
DAPI
100 µm

Figure S3

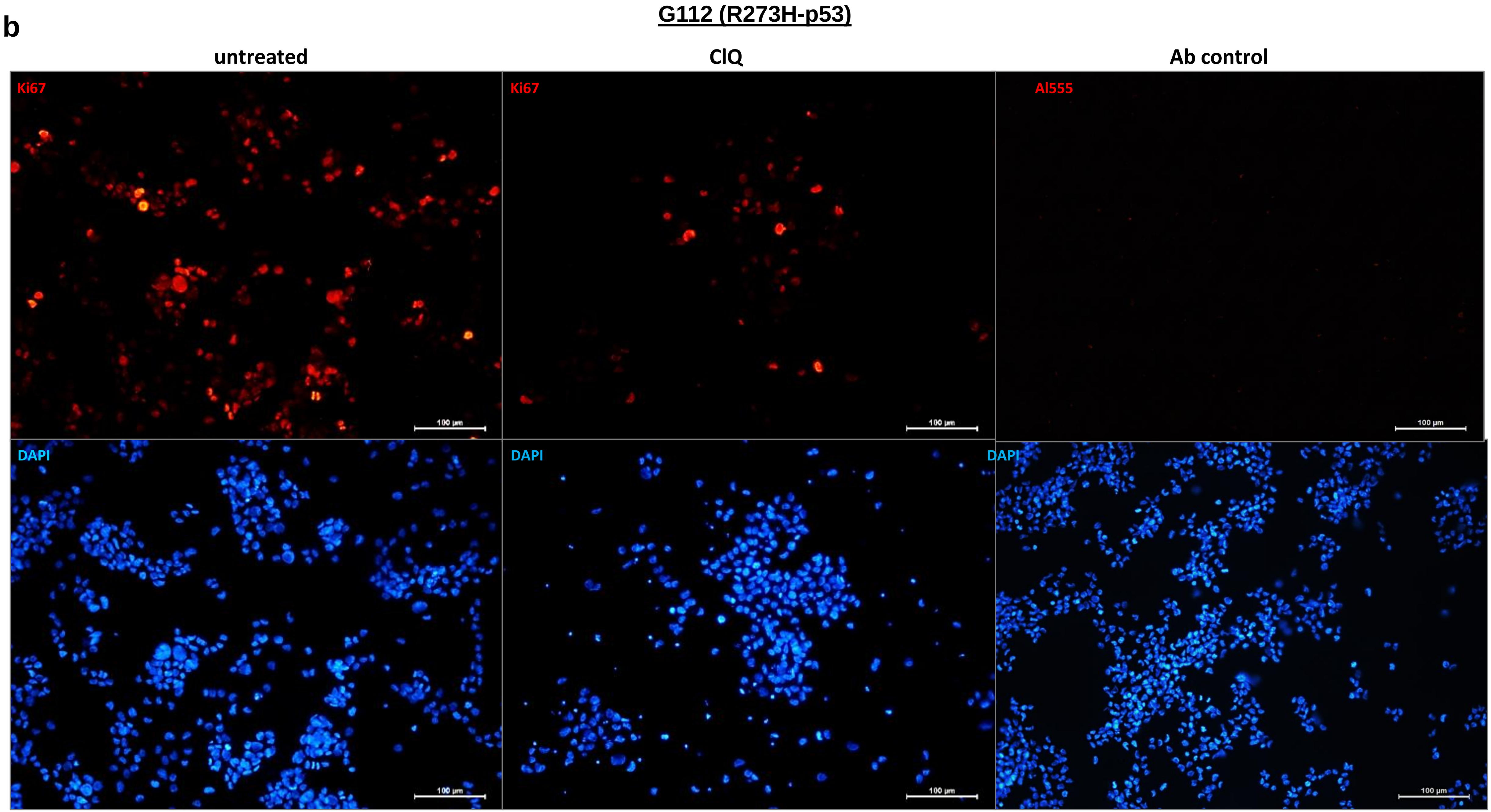
b
G112 (R273H-p53)
untreated
ClQ
Ab control
Ki67
Ki67
Al555
DAPI
DAPI
DAPI
100 µm

Figure S3

#1095 (p53 null)

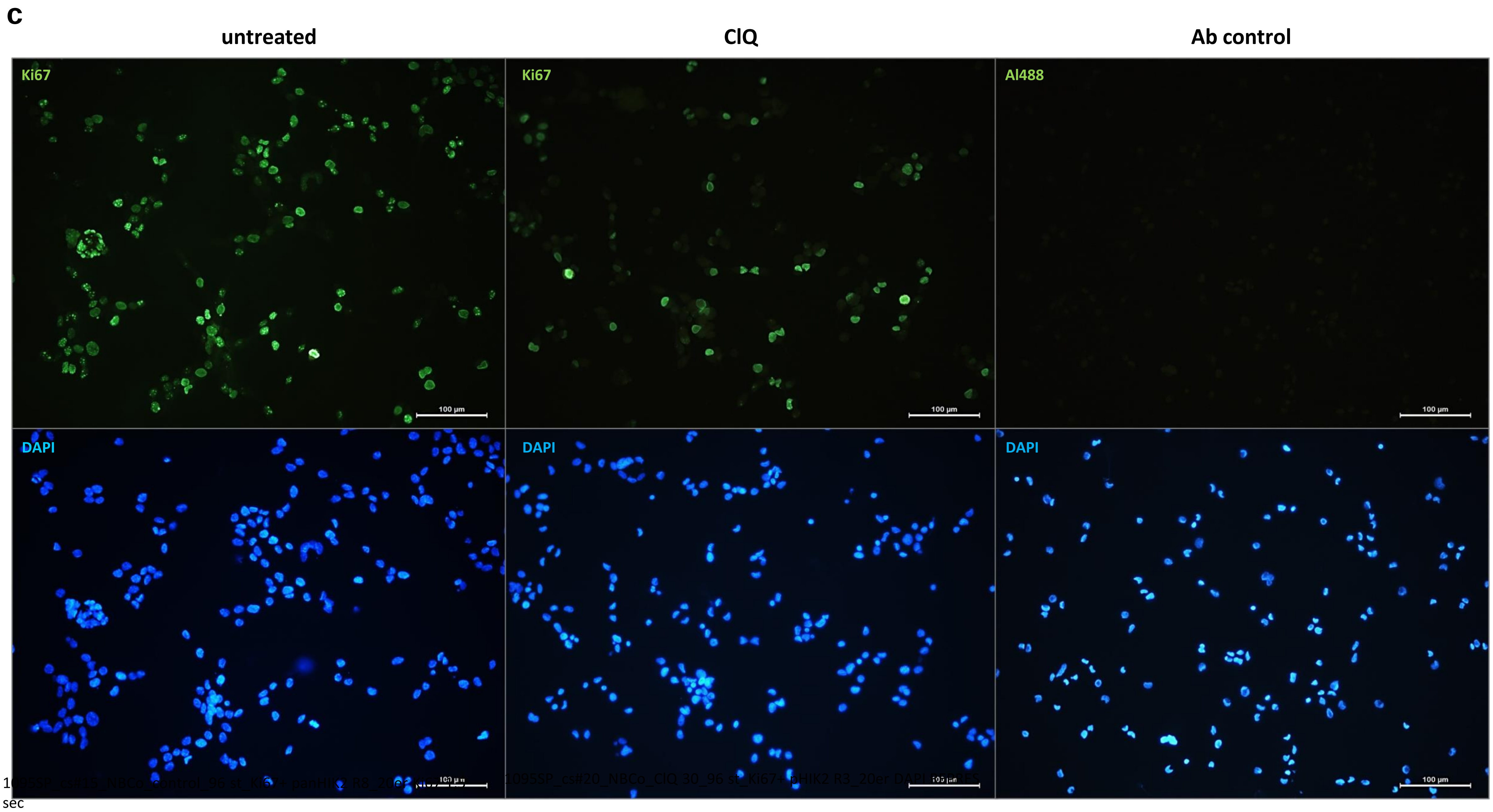

Figure S3

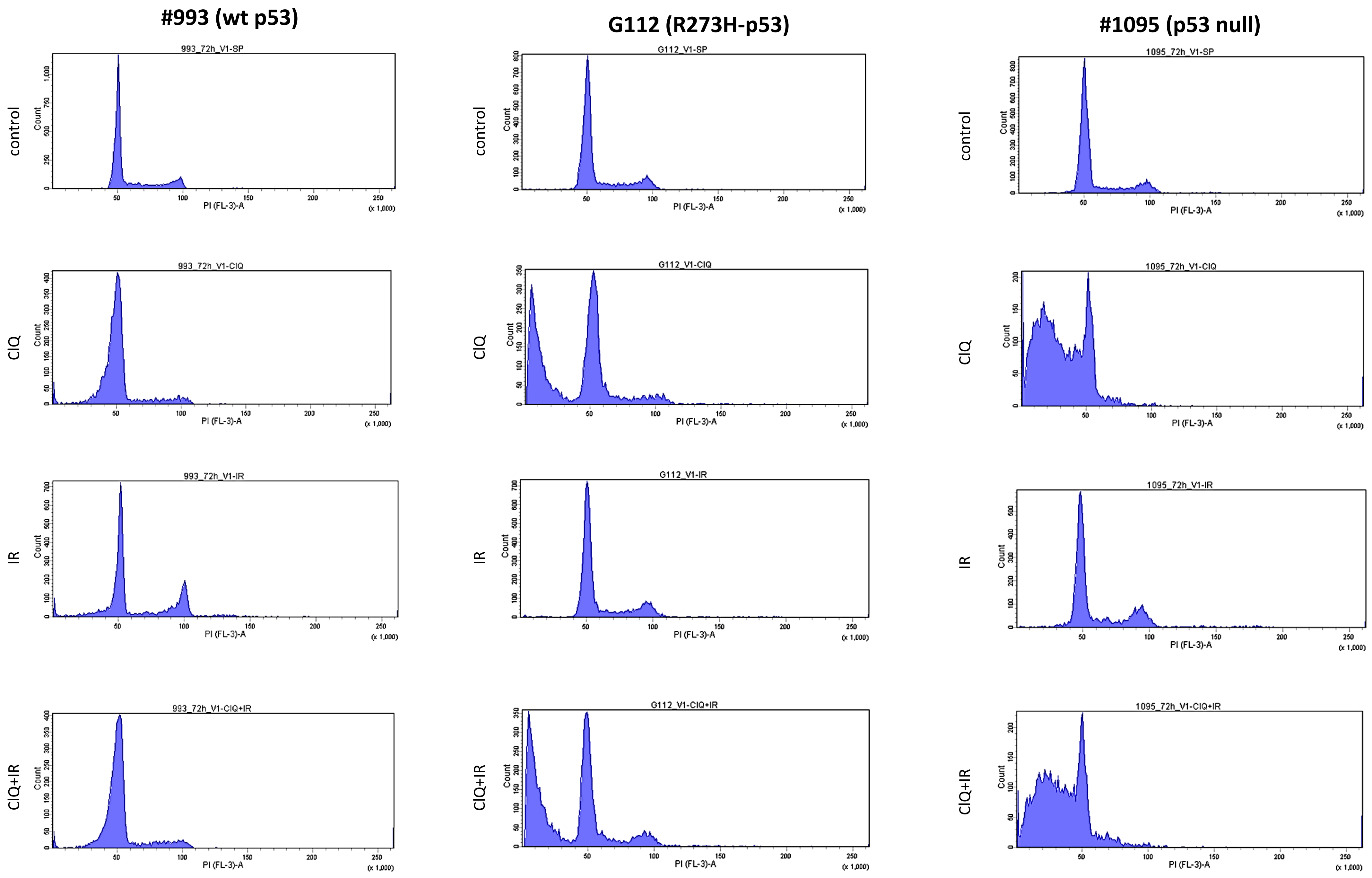

#993 (wt p53)
G112 (R273H-p53)
#1095 (p53 null)
control
ClQ
IR
ClQ+IR
993_72h_V1-SP
G112_V1-SP
1095_72h_V1-SP
993_72h_V1-ClQ
G112_V1-ClQ
1095_72h_V1-ClQ
993_72h_V1-IR
G112_V1-IR
1095_72h_V1-IR
993_72h_V1-ClQ+IR
G112_V1-ClQ+IR
1095_72h_V1-ClQ+IR
Count
PI (FL-3)-A
(x 1,000)

Figure S4

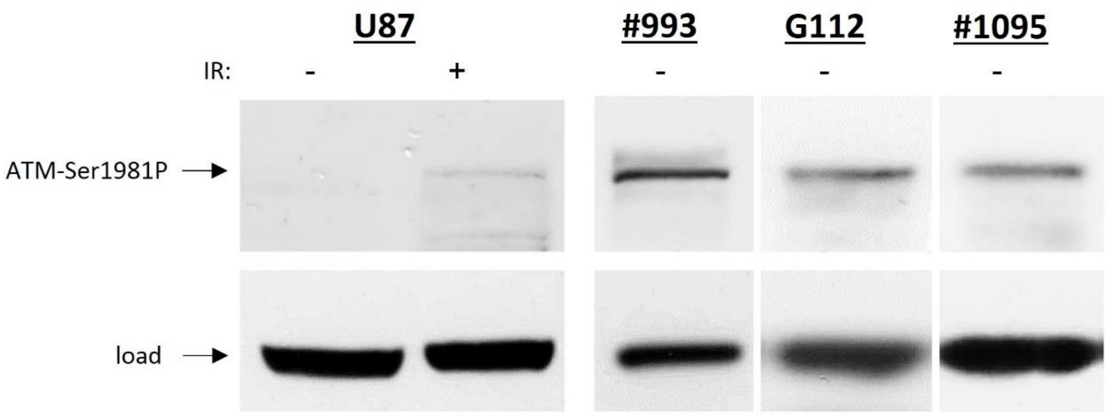

Figure S5

a

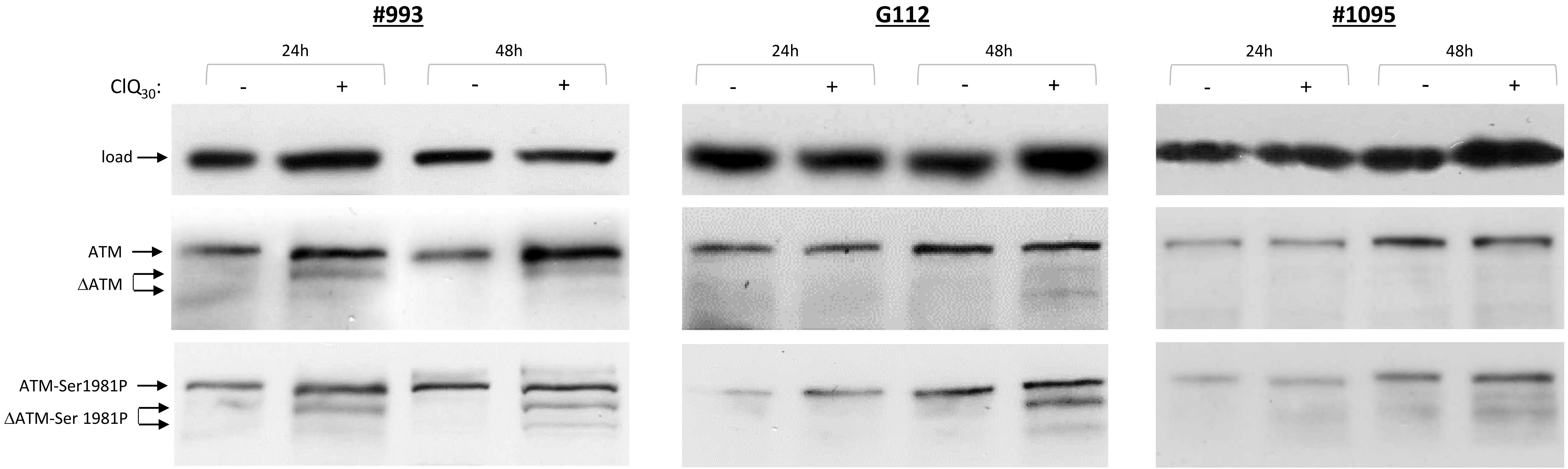
#993
G112
#1095
24h
48h
ClQ30:
-
+
load
ATM
ΔATM
ATM-Ser1981P
ΔATM-Ser 1981P

Figure S6

b

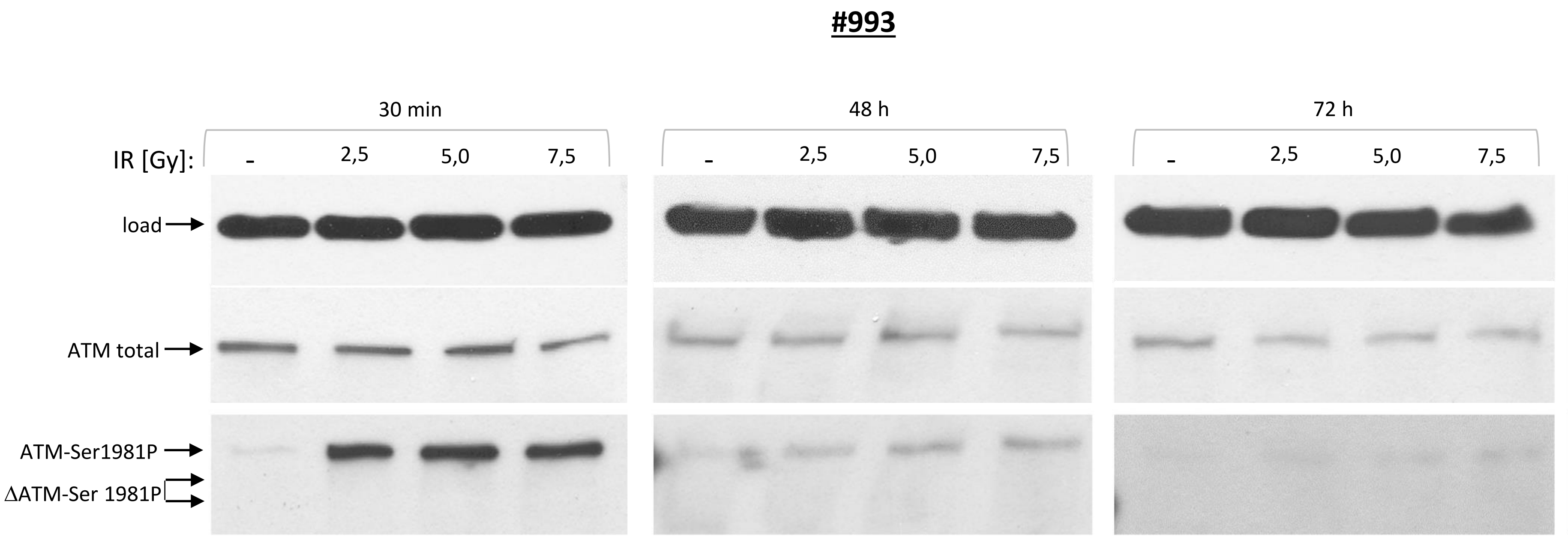
#993
30 min
48 h
72 h
IR [Gy]:
-
2,5
5,0
7,5
load
ATM total
ATM-Ser1981P
ΔATM-Ser 1981P

Figure S6

a

ClQ_DEGs_#993 (wtp53)

Probe	Symbol	Description	logFC #993 ClQ vs untreated	P.Value #993 ClQ vs untreated	FDR #993 ClQ vs untreated
10024_at	TROAP	trophinin associated protein (tast	-1,089224694	1,12E-008	3,41E-006
10112_at	KIF20A	kinesin family member 20A	-1,51878522	2,22E-010	2,59E-007
1033_at	CDKN3	cyclin-dependent kinase inhibitor	-1,10629628	1,04E-008	3,25E-006
10371_at	SEMA3A	sema domain, immunoglobulin d	-1,371386841	2,95E-008	6,64E-006
10403_at	NDC80	NDC80 homolog, kinetochore co	-1,479461246	3,28E-009	1,48E-006
10615_at	SPAG5	sperm associated antigen 5	-1,125836192	1,09E-009	7,98E-007
1062_at	CENPE	centromere protein E, 312kDa	-1,426468934	4,65E-008	9,31E-006
10635_at	RAD51AP1	RAD51 associated protein 1	-1,327058656	6,99E-008	1,34E-005
10682_at	EBP	emopamil binding protein (sterol	1,25911264	8,17E-010	6,74E-007
10721_at	POLQ	polymerase (DNA directed), thet	-1,266294243	2,04E-007	3,14E-005
10733_at	PLK4	polo-like kinase 4 (Drosophila)	-1,250013612	4,05E-008	8,62E-006
11004_at	KIF2C	kinesin family member 2C	-1,34467197	3,60E-009	1,53E-006
11065_at	UBE2C	ubiquitin-conjugating enzyme E2	-1,077519291	3,40E-009	1,50E-006
11113_at	CIT	citron (rho-interacting, serine/thre	-1,058557873	2,25E-007	3,38E-005
11161_at	C14orf1	chromosome 14 open reading fra	1,119682631	5,08E-008	1,00E-005
113115_at	FAM54A	family with sequence similarity 5	-1,043804999	1,83E-007	2,87E-005
1410_at	CRYAB	crystallin, alpha B	1,026350939	7,61E-009	2,69E-006
146956_at	EME1	essential meiotic endonuclease	-1,145995374	1,12E-007	1,94E-005
150221_at	RIMBP3C	RIMS binding protein 3C	-1,42153587	0,000947503	0,035251727
150468_at	CKAP2L	cytoskeleton associated protein	-1,450643508	8,81E-011	1,59E-007
151246_at	SGOL2	shugoshin-like 2 (S. pombe)	-1,538298644	3,75E-010	4,12E-007
151648_at	SGOL1	shugoshin-like 1 (S. pombe)	-1,302146493	1,51E-008	4,20E-006
157313_at	CDCA2	cell division cycle associated 2	-1,295357771	4,49E-008	9,16E-006
157570_at	ESCO2	establishment of cohesion 1 hom	-1,598861831	2,75E-008	6,39E-006
158471_at	PRUNE2	prune homolog 2 (Drosophila)	1,207525049	8,07E-009	2,75E-006
1717_at	DHCR7	7-dehydrocholesterol reductase	1,347347924	2,24E-011	6,32E-008
1718_at	DHCR24	24-dehydrocholesterol reductase	1,001865609	5,26E-008	1,03E-005
1763_at	DNA2	DNA replication helicase 2 homo	-1,02678217	2,87E-006	0,000290317

Figure S7

b

Probe	Symbol	Description	logFC #993 ClQ vs untreated	P.Value #993 ClQ vs untreated	FDR #993 ClQ vs untreated
2170_at	FABP3	fatty acid binding protein 3, musc	2,005550665	2,42E-009	1,24E-006
2177_at	FANCD2	Fanconi anemia, complementatio	-1,160062695	8,79E-008	1,55E-005
2187_at	FANCB	Fanconi anemia, complementatio	-1,319742454	7,34E-007	9,50E-005
2194_at	FASN	fatty acid synthase	1,199029246	2,02E-008	5,23E-006
219790_at	RTKN2	rhotekin 2	-1,58672524	6,94E-008	1,34E-005
220042_at	C11orf82	chromosome 11 open reading fra	-1,059750794	1,49E-006	0,000176055
220134_at	C18orf24	chromosome 18 open reading fra	-1,159800013	1,21E-008	3,54E-006
221150_at	C13orf3	chromosome 13 open reading fra	-1,304631264	8,56E-009	2,82E-006
2222_at	FDFT1	farnesyl-diphosphate farnesyltran	1,055670855	8,54E-010	6,76E-007
2224_at	FDPS	farnesyl diphosphate synthase (f	1,339085985	3,54E-012	2,33E-008
22974_at	TPX2	TPX2, microtubule-associated, h	-1,208617691	5,11E-010	4,92E-007
22995_at	CEP152	centrosomal protein 152kDa	-1,224930239	6,91E-007	9,11E-005
23175_at	LPIN1	lipin 1	1,154855364	1,15E-008	3,45E-006
23397_at	NCAPH	non-SMC condensin I complex, s	-1,124297825	2,44E-008	5,90E-006
24137_at	KIF4A	kinesin family member 4A	-1,364296701	2,92E-008	6,64E-006
2494_at	NR5A2	nuclear receptor subfamily 5, gro	-1,074243736	5,32E-007	7,17E-005
255394_at	TCP11L2	t-complex 11 (mouse)-like 2	1,207017902	2,79E-008	6,41E-006
259266_at	ASPM	asp (abnormal spindle) homolog,	-1,374532969	7,55E-009	2,69E-006
283431_at	GAS2L3	growth arrest-specific 2 like 3	-1,362695449	1,56E-009	9,66E-007
2846_at	LPAR4	lysophosphatidic acid receptor 4	-1,117705621	2,58E-005	0,00198455
284992_at	CCDC150	coiled-coil domain containing 150	-1,377118834	2,37E-009	1,24E-006
286151_at	FBXO43	F-box protein 43	-1,181930841	2,04E-008	5,23E-006
29127_at	RACGAP1	Rac GTPase activating protein 1	-1,072090453	1,72E-008	4,73E-006
3161_at	HMMR	hyaluronan-mediated motility rec	-1,124591612	1,43E-007	2,36E-005
343099_at	CCDC18	coiled-coil domain containing 18	-1,103023973	1,30E-007	2,21E-005
347240_at	KIF24	kinesin family member 24	-1,001927841	1,91E-007	2,98E-005
347731_at	LRRTM3	leucine rich repeat transmembra	-1,054923233	4,63E-008	9,31E-006
3638_at	INSIG1	insulin induced gene 1	1,444487764	1,39E-011	4,75E-008
375444_at	C5orf34	chromosome 5 open reading fran	-1,088822218	5,72E-007	7,60E-005
3832_at	KIF11	kinesin family member 11	-1,156423099	7,04E-007	9,17E-005
39_at	ACAT2	acetyl-Coenzyme A acetyltransfe	1,274906128	1,93E-010	2,38E-007

Figure S7

c

Probe	Symbol	Description	logFC #993 ClQ vs untreated	P.Value #993 ClQ vs untreated	FDR #993 ClQ vs untreated
4047_at	LSS	lanosterol synthase (2,3-oxidosc	1,122695118	3,54E-008	7,61E-006
4085_at	MAD2L1	MAD2 mitotic arrest deficient-lik	-1,45495862	1,75E-008	4,76E-006
4288_at	MKI67	antigen identified by monoclonal	-1,122972876	7,75E-008	1,43E-005
4597_at	MVD	mevalonate (diphospho) decarbo	1,132866547	7,88E-010	6,74E-007
4603_at	MYBL1	v-myb myeloblastosis viral oncog	-1,160321916	3,89E-007	5,61E-005
4751_at	NEK2	NIMA (never in mitosis gene a)-r	-1,260741692	2,44E-009	1,24E-006
50814_at	NSDHL	NAD(P) dependent steroid dehyd	1,253678078	3,94E-011	9,74E-008
51203_at	NUSAP1	nucleolar and spindle associated	-1,269184356	7,87E-009	2,73E-006
51478_at	HSD17B7	hydroxysteroid (17-beta) dehydro	1,222537624	0,00013483	0,00760309
51512_at	GTSE1	G-2 and S-phase expressed 1	-1,143496205	1,21E-008	3,54E-006
53353_at	LRP1B	low density lipoprotein-related pr	1,022647953	4,71E-007	6,66E-005
5347_at	PLK1	polo-like kinase 1 (Drosophila)	-1,24526622	1,34E-009	8,55E-007
54443_at	ANLN	anillin, actin binding protein	-1,388659462	1,06E-009	7,98E-007
54478_at	FAM64A	family with sequence similarity 6	-1,228741633	8,95E-008	1,57E-005
54821_at	ERCC6L	excision repair cross-complemen	-1,615998378	2,03E-008	5,23E-006
54873_at	PALMD	palmdelphin	-1,026847857	4,90E-007	6,83E-005
54892_at	NCAPG2	non-SMC condensin II complex,	-1,055511832	5,54E-009	2,07E-006
55010_at	C12orf48	chromosome 12 open reading fra	-1,378058059	8,46E-008	1,51E-005
55143_at	CDCA8	cell division cycle associated 8	-1,201909605	1,19E-006	0,000147179
55215_at	FANCI	Fanconi anemia, complementati	-1,0866537	5,07E-007	6,97E-005
55247_at	NEIL3	nei endonuclease VIII-like 3 (E. c	-1,474875965	2,26E-009	1,24E-006
55320_at	C14orf106	chromosome 14 open reading fra	-1,181587058	8,28E-008	1,50E-005
55329_at	MNS1	meiosis-specific nuclear structur	-1,351894559	1,28E-007	2,19E-005
55345_at	C4orf21	chromosome 4 open reading fran	-1,055603862	1,27E-006	0,000154107
55355_at	HJURP	Holliday junction recognition prot	-1,190856709	7,39E-009	2,69E-006
55388_at	MCM10	minichromosome maintenance c	-1,034739786	2,76E-006	0,000282633
55635_at	DEPDC1	DEP domain containing 1	-1,203446411	1,39E-007	2,32E-005
55771_at	PRR11	proline rich 11	-1,202912243	3,88E-009	1,54E-006
55789_at	DEPDC1B	DEP domain containing 1B	-1,244595019	9,81E-009	3,13E-006
55872_at	PBK	PDZ binding kinase	-1,601857891	3,90E-009	1,54E-006

Figure S7

d

Probe	Symbol	Description	logFC #993 ClQ vs untreated	P.Value #993 ClQ vs untreated	FDR #993 ClQ vs untreated
55902_at	ACSS2	acyl-CoA synthetase short-chair	1,270824058	1,24E-010	1,98E-007
56992_at	KIF15	kinesin family member 15	-1,473551451	3,04E-008	6,75E-006
57082_at	CASC5	cancer susceptibility candidate 5	-1,493793006	2,72E-008	6,39E-006
57405_at	SPC25	SPC25, NDC80 kinetochore com	-1,472807972	5,28E-012	2,61E-008
57650_at	KIAA1524	KIAA1524	-1,209168964	4,33E-008	8,95E-006
5983_at	RFC3	replication factor C (activator 1) 3	-1,054531028	2,69E-008	6,39E-006
6241_at	RRM2	ribonucleotide reductase M2 poly	-1,138488224	1,05E-008	3,25E-006
6307_at	SC4MOL	sterol-C4-methyl oxidase-like	1,17905442	1,80E-008	4,81E-006
6319_at	SCD	stearoyl-CoA desaturase (delta-9	1,275894886	2,80E-014	5,53E-010
64105_at	CENPK	centromere protein K	-1,08765816	2,33E-008	5,69E-006
64151_at	NCAPG	non-SMC condensin I complex, s	-1,274424255	2,08E-009	1,20E-006
6491_at	STIL	SCL/TAL1 interrupting locus	-1,14454901	1,38E-008	3,95E-006
6565_at	SLC15A2	solute carrier family 15 (H+/pepti	-1,047785742	1,00E-005	0,000872656
675_at	BRCA2	breast cancer 2, early onset	-1,013227035	1,22E-006	0,000150047
677807_at	SNORA22	small nucleolar RNA, H/ACA box	1,071728985	0,001066584	0,038037664
6790_at	AURKA	aurora kinase A	-1,125210189	8,51E-009	2,82E-006
699_at	BUB1	budding uninhibited by benzimida	-1,408561792	1,29E-009	8,48E-007
7018_at	TF	transferrin	1,623492994	4,35E-009	1,69E-006
701_at	BUB1B	budding uninhibited by benzimida	-1,433215512	6,59E-011	1,30E-007
7108_at	TM7SF2	transmembrane 7 superfamily m	1,339665049	5,01E-010	4,92E-007
7143_at	TNR	tenascin R (restrictin, janusin)	-1,140439309	1,69E-005	0,001392452
7153_at	TOP2A	topoisomerase (DNA) II alpha 17	-1,222894022	2,11E-009	1,20E-006
7272_at	TTK	TTK protein kinase	-1,707133652	1,59E-010	2,10E-007
7516_at	XRCC2	X-ray repair complementing defe	-1,011422703	7,40E-007	9,51E-005
79071_at	ELOVL6	ELOVL family member 6, elonga	1,003205781	3,96E-007	5,68E-005
79733_at	E2F8	E2F transcription factor 8	-1,302816466	1,98E-009	1,19E-006
79801_at	SHCBP1	SHC SH2-domain binding protein	-1,219004921	3,64E-009	1,53E-006
7984_at	ARHGEF5	Rho guanine nucleotide exchang	1,590642658	0,000468802	0,02071204
79866_at	C13orf34	chromosome 13 open reading fra	-1,486829361	6,54E-011	1,30E-007
81624_at	DIAPH3	diaphanous homolog 3 (Drosoph	-1,173999252	2,26E-007	3,38E-005
81930_at	KIF18A	kinesin family member 18A	-1,540900917	3,36E-008	7,30E-006

Figure S7

e

Probe	Symbol	Description	logFC #993 ClQ vs untreated	P.Value #993 ClQ vs untreated	FDR #993 ClQ vs untreated
8342_at	HIST1H2BM	histone cluster 1, H2bm	-1,053868785	2,55E-005	0,001967854
83461_at	CDCA3	cell division cycle associated 3	-1,433633318	2,68E-012	2,33E-008
83540_at	NUF2	NUF2, NDC80 kinetochore comp	-1,310186527	2,53E-009	1,25E-006
83990_at	BRIP1	BRCA1 interacting protein C-terr	-1,034453779	2,19E-006	0,000240308
84824_at	FCRLA	Fc receptor-like A	1,158022788	1,40E-010	1,98E-007
890_at	CCNA2	cyclin A2	-1,254531232	2,72E-009	1,28E-006
891_at	CCNB1	cyclin B1	-1,503325343	1,44E-011	4,75E-008
89839_at	ARHGAP11E	Rho GTPase activating protein 1	-1,489673573	8,84E-009	2,87E-006
9055_at	PRC1	protein regulator of cytokinesis 1	-1,235860321	2,73E-009	1,28E-006
9133_at	CCNB2	cyclin B2	-1,239027552	4,34E-008	8,95E-006
9134_at	CCNE2	cyclin E2	-1,042230477	1,25E-006	0,000152591
9156_at	EXO1	exonuclease 1	-1,254033153	3,58E-007	5,21E-005
92211_at	PCDH21	protocadherin 21	1,146903181	1,16E-009	8,23E-007
9232_at	PTTG1	pituitary tumor-transforming 1	-1,110943355	7,05E-008	1,34E-005
9415_at	FADS2	fatty acid desaturase 2	1,055782386	2,79E-009	1,29E-006
9493_at	KIF23	kinesin family member 23	-1,427337386	1,38E-010	1,98E-007
9585_at	KIF20B	kinesin family member 20B	-1,270617743	2,68E-007	3,96E-005
9768_at	KIAA0101	KIAA0101	-1,144136781	4,81E-007	6,75E-005
9787_at	DLGAP5	discs, large (Drosophila) homolo	-1,520557272	1,22E-009	8,32E-007
9824_at	ARHGAP11A	Rho GTPase activating protein 1	-1,181800944	5,43E-007	7,26E-005
9833_at	MELK	maternal embryonic leucine zipp	-1,076852765	4,30E-008	8,95E-006
983_at	CDC2	cell division cycle 2, G1 to S and	-1,563353634	7,09E-010	6,38E-007
9928_at	KIF14	kinesin family member 14	-1,401389389	3,81E-009	1,54E-006
995_at	CDC25C	cell division cycle 25 homolog C	-1,261221854	1,49E-008	4,20E-006

Figure S7

a

ClQ_DEGs_G112 (R273H)

Probe	Symbol	Description	logFC G112 ClQ vs untr	P.Value G112 ClQ vs untr	FDR G112 ClQ vs untr
100033805_at	SNORD115-31	small nucleolar RNA, C/D box 115-31	-0,711657548	1,96E-006	6,54E-005
10007_at	GNPDA1	glucosamine-6-phosphate deaminase 1	0,772892	5,71E-012	1,53E-009
100129196_at	LOC100129196	similar to hCG2033298	-0,616059764	7,91E-005	0,001399894
100129762_at	LOC100129762	similar to KIAA0367	1,103630612	5,42E-015	4,13E-012
100133188_at	LOC100133364	hypothetical LOC100133364	0,91738411	3,21E-014	1,99E-011
10085_at	EDIL3	EGF-like repeats and discoidin I-like domains 3	-1,069723465	4,65E-015	3,68E-012
10112_at	KIF20A	kinesin family member 20A	-0,704770275	3,55E-011	6,75E-009
10253_at	SPRY2	sprouty homolog 2 (Drosophila)	-0,946604911	2,78E-014	1,83E-011
10371_at	SEMA3A	sema domain, immunoglobulin domain (Ig), short b	-0,734522097	8,19E-012	2,11E-009
10437_at	IFI30	interferon, gamma-inducible protein 30	1,079246586	1,21E-013	5,30E-011
10457_at	GPNMB	glycoprotein (transmembrane) nmb	2,311213114	6,03E-023	1,19E-018
10493_at	VAT1	vesicle amine transport protein 1 homolog (T. califo	0,663255905	2,21E-010	3,24E-008
10577_at	NPC2	Niemann-Pick disease, type C2	1,004417609	1,18E-013	5,30E-011
10769_at	PLK2	polo-like kinase 2 (Drosophila)	-0,585759398	1,31E-009	1,40E-007
10891_at	PPARGC1A	peroxisome proliferator-activated receptor gamma,	0,698675129	2,52E-012	7,67E-010
11010_at	GLIPR1	GLI pathogenesis-related 1	-0,860821051	5,12E-014	2,60E-011
1116_at	CHI3L1	chitinase 3-like 1 (cartilage glycoprotein-39)	-0,646882965	3,46E-011	6,75E-009
112770_at	C1orf85	chromosome 1 open reading frame 85	0,585853019	1,38E-010	2,17E-008
113146_at	AHNAK2	AHNAK nucleoprotein 2	0,596022265	5,59E-009	4,81E-007
114907_at	FBXO32	F-box protein 32	1,153858498	9,41E-016	1,02E-012
116496_at	FAM129A	family with sequence similarity 129, member A	0,776804015	1,54E-012	4,83E-010
1183_at	CLCN4	chloride channel 4	0,592528217	3,04E-010	4,23E-008
1186_at	CLCN7	chloride channel 7	0,758483866	4,17E-011	7,72E-009
1193_at	CLIC2	chloride intracellular channel 2	0,762183175	5,06E-012	1,39E-009
1200_at	TPP1	tripeptidyl peptidase I	1,190330849	9,33E-017	2,05E-013
1244_at	ABCC2	ATP-binding cassette, sub-family C (CFTR/MRP),	0,630896828	1,07E-012	3,60E-010
128344_at	C1orf88	chromosome 1 open reading frame 88	0,653802258	9,36E-010	1,06E-007
128637_at	TBC1D20	TBC1 domain family, member 20	0,638969224	2,47E-013	1,00E-010
134429_at	STARD4	StAR-related lipid transfer (START) domain contair	0,872170769	5,87E-013	2,08E-010

Figure S8

b

Probe	Symbol	Description	logFC G112 ClQ vs untr	P.Value G112 ClQ vs untr	FDR G112 ClQ vs untr
138639_at	PTPDC1	protein tyrosine phosphatase domain containing 1	0,893940432	1,88E-015	1,77E-012
1435_at	CSF1	colony stimulating factor 1 (macrophage)	0,807308891	8,32E-014	4,02E-011
1491_at	CTH	cystathionase (cystathionine gamma-lyase)	0,699884596	2,55E-011	5,43E-009
1497_at	CTNS	cystinosis, nephropathic	0,612474241	2,51E-009	2,42E-007
1509_at	CTSD	cathepsin D	0,62345171	4,59E-011	8,33E-009
153222_at	C5orf41	chromosome 5 open reading frame 41	0,610044575	7,25E-009	6,01E-007
158471_at	PRUNE2	prune homolog 2 (Drosophila)	1,095021586	2,23E-016	4,02E-013
162394_at	SLFN5	schlafen family member 5	1,087347333	2,90E-016	4,42E-013
1649_at	DDIT3	DNA-damage-inducible transcript 3	1,608247203	2,40E-019	1,19E-015
1717_at	DHCR7	7-dehydrocholesterol reductase	0,800342565	1,02E-014	7,18E-012
1848_at	DUSP6	dual specificity phosphatase 6	-1,439855174	7,01E-018	2,31E-014
1958_at	EGR1	early growth response 1	-1,025198827	9,76E-016	1,02E-012
202374_at	STK32A	serine/threonine kinase 32A	0,737348076	3,94E-010	5,28E-008
2035_at	EPB41	erythrocyte membrane protein band 4.1 (elliptocyt	0,7715115	1,00E-012	3,43E-010
2113_at	ETS1	v-ets erythroblastosis virus E26 oncogene homolog	0,611114762	1,90E-012	5,86E-010
2118_at	ETV4	ets variant gene 4 (E1A enhancer binding protein, I	-1,106373552	8,68E-017	2,05E-013
2119_at	ETV5	ets variant gene 5 (ets-related molecule)	-0,850582169	1,13E-013	5,30E-011
2180_at	ACSL1	acyl-CoA synthetase long-chain family member 1	0,871186309	4,42E-012	1,25E-009
218_at	ALDH3A1	aldehyde dehydrogenase 3 family, memberA1	-0,688470108	3,21E-011	6,49E-009
2194_at	FASN	fatty acid synthase	1,073975374	2,15E-014	1,46E-011
2196_at	FAT2	FAT tumor suppressor homolog 2 (Drosophila)	-1,169895931	9,20E-016	1,02E-012
2222_at	FDFT1	farnesyl-diphosphate farnesyltransferase 1	0,672728934	4,43E-012	1,25E-009
222484_at	LNX2	ligand of numb-protein X 2	0,595088032	5,21E-010	6,69E-008
2224_at	FDPS	farnesyl diphosphate synthase (farnesyl pyrophosp	0,764947299	1,37E-011	3,15E-009
2239_at	GPC4	glypican 4	-0,722340733	1,15E-009	1,25E-007
22822_at	PHLDA1	pleckstrin homology-like domain, family A, membe	-0,662269737	8,84E-012	2,24E-009
22885_at	ABLIM3	actin binding LIM protein family, member 3	0,90697846	4,36E-014	2,33E-011
23007_at	PLCH1	phospholipase C, eta 1	-0,810270617	9,92E-011	1,61E-008
23175_at	LPIN1	lipin 1	1,230766508	1,20E-018	4,74E-015
2353_at	FOS	v-fos FBJ murine osteosarcoma viral oncogene hor	-0,660050583	6,52E-010	7,96E-008

Figure S8

c

Probe	Symbol	Description	logFC G112 ClQ vs untr	P.Value G112 ClQ vs untr	FDR G112 ClQ vs untr
23645_at	PPP1R15A	protein phosphatase 1, regulatory (inhibitor) subun	0,667043088	1,01E-009	1,12E-007
24137_at	KIF4A	kinesin family member 4A	-0,585464805	2,48E-007	1,14E-005
2512_at	FTL	ferritin, light polypeptide	0,950172466	2,30E-008	1,57E-006
254958_at	REXO1L1	REX1, RNA exonuclease 1 homolog (S. cerevisiae	-0,824506223	0,001323898	0,012665015
255394_at	TCP11L2	t-complex 11 (mouse)-like 2	0,749514758	7,26E-011	1,23E-008
255743_at	NPNT	nephronectin	-0,608863384	3,75E-011	7,00E-009
255928_at	SYT14	synaptotagmin XIV	0,610114951	7,89E-010	9,30E-008
256987_at	SERINC5	serine incorporator 5	0,848234005	2,83E-012	8,50E-010
25925_at	ZNF521	zinc finger protein 521	-0,70464454	5,68E-011	1,01E-008
25928_at	SOSTDC1	sclerostin domain containing 1	1,11510801	1,20E-013	5,30E-011
26278_at	SACS	spastic ataxia of Charlevoix-Saguenay (sacsin)	-0,627395584	6,21E-010	7,68E-008
2669_at	GEM	GTP binding protein overexpressed in skeletal mus	1,007800216	7,45E-015	5,46E-012
2673_at	GFPT1	glutamine-fructose-6-phosphate transaminase 1	0,608666162	1,57E-011	3,49E-009
27303_at	RBMS3	RNA binding motif, single stranded interacting prot	-0,635668305	4,44E-010	5,86E-008
2799_at	GNS	glucosamine (N-acetyl)-6-sulfatase	0,675557754	8,49E-010	9,83E-008
2805_at	GOT1	glutamic-oxaloacetic transaminase 1, soluble (asp	0,790836376	1,65E-010	2,55E-008
2835_at	GPR12	G protein-coupled receptor 12	-0,595811745	4,65E-006	0,000137228
28526_at	TRDC	T cell receptor delta constant	-1,100143064	3,49E-014	2,03E-011
285605_at	DTWD2	DTW domain containing 2	0,978515203	8,66E-013	3,01E-010
285888_at	CNPY1	canopy 1 homolog (zebrafish)	-0,630820844	5,96E-011	1,03E-008
287_at	ANK2	ankyrin 2, neuronal	0,663831171	8,75E-010	1,01E-007
28951_at	TRIB2	tribbles homolog 2 (Drosophila)	-0,741080095	9,76E-011	1,61E-008
2896_at	GRN	granulin	0,762058638	3,64E-011	6,87E-009
3037_at	HAS2	hyaluronan synthase 2	-0,6196211	2,55E-010	3,66E-008
3073_at	HEXA	hexosaminidase A (alpha polypeptide)	0,812087917	1,85E-013	7,80E-011
3156_at	HMGCR	3-hydroxy-3-methylglutaryl-Coenzyme A reductase	0,694954585	2,04E-010	3,08E-008
3157_at	HMGCS1	3-hydroxy-3-methylglutaryl-Coenzyme A synthase	0,779985102	1,34E-013	5,78E-011
3162_at	HMOX1	heme oxygenase (decycling) 1	0,8145864	2,90E-011	6,00E-009
3304_at	HSPA1B	heat shock 70kDa protein 1B	0,665467775	2,39E-008	1,61E-006
3371_at	TNC	tenascin C	0,727636826	4,70E-014	2,45E-011
340529_at	PABPC1L2A	poly(A) binding protein, cytoplasmic 1-like 2A	-0,753450827	9,70E-005	0,001650875

Figure S8

d

Probe	Symbol	Description	logFC G112 ClQ vs untr	P.Value G112 ClQ vs untr	FDR G112 ClQ vs untr
3417_at	IDH1	isocitrate dehydrogenase 1 (NADP), soluble	0,839432429	5,34E-012	1,45E-009
347735_at	SERINC2	serine incorporator 2	0,714313465	1,74E-011	3,79E-009
3638_at	INSIG1	insulin induced gene 1	0,906294292	3,61E-014	2,04E-011
3782_at	KCNN3	potassium intermediate/small conductance calciur	-0,589982612	8,47E-009	6,87E-007
387758_at	FIBIN	fin bud initiation factor	0,594456774	3,15E-007	1,40E-005
3934_at	LCN2	lipocalin 2	0,714444612	2,29E-009	2,24E-007
3955_at	LFNG	LFNG O-fucosylpeptide 3-beta-N-acetylglucosamir	-0,760487235	1,14E-011	2,75E-009
401190_at	RGS7BP	regulator of G-protein signaling 7 binding protein	0,613849867	1,03E-007	5,55E-006
4047_at	LSS	lanosterol synthase (2,3-oxidosqualene-lanosterol	1,047239054	1,74E-016	3,44E-013
4071_at	TM4SF1	transmembrane 4 L six family member 1	-0,705102317	4,70E-013	1,72E-010
4074_at	M6PR	mannose-6-phosphate receptor (cation dependent)	0,594166277	1,01E-009	1,12E-007
4323_at	MMP14	matrix metallopeptidase 14 (membrane-inserted)	0,740108868	1,17E-012	3,85E-010
440015_at	LOC440015	similar to double homeobox, 4	-0,614689558	4,56E-005	0,000901033
467_at	ATF3	activating transcription factor 3	0,596587984	6,17E-011	1,05E-008
4688_at	NCF2	neutrophil cytosolic factor 2	0,704383173	2,71E-011	5,70E-009
4758_at	NEU1	sialidase 1 (lysosomal sialidase)	1,251878833	2,81E-016	4,42E-013
4779_at	NFE2L1	nuclear factor (erythroid-derived 2)-like 1	0,749653515	1,93E-011	4,16E-009
482_at	ATP1B2	ATPase, Naسخ� transporting, beta 2 polypeptide	-0,64060874	9,19E-012	2,27E-009
4856_at	NOV	nephroblastoma overexpressed gene	1,720341681	3,97E-020	2,62E-016
4919_at	ROR1	receptor tyrosine kinase-like orphan receptor 1	1,02787474	7,56E-017	2,05E-013
50650_at	ARHGEF3	Rho guanine nucleotide exchange factor (GEF) 3	0,589473809	2,72E-009	2,61E-007
5184_at	PEPD	peptidase D	0,652830475	3,31E-011	6,63E-009
5208_at	PFKFB2	6-phosphofructo-2-kinase/fructose-2,6-biphosphata	0,840089563	1,18E-011	2,77E-009
5327_at	PLAT	plasminogen activator, tissue	-1,878928756	1,55E-022	1,53E-018
5337_at	PLD1	phospholipase D1, phosphatidylcholine-specific	-0,629838907	1,16E-011	2,76E-009
54020_at	SLC37A1	solute carrier family 37 (glycerol-3-phosphate trans	0,62960635	2,35E-009	2,29E-007
54557_at	SGTB	small glutamine-rich tetratricopeptide repeat (TPR)	0,891727925	1,30E-011	3,02E-009
5476_at	CTSA	cathepsin A	0,656097847	4,25E-013	1,62E-010
54800_at	KLHL24	kelch-like 24 (Drosophila)	1,055183409	7,40E-016	9,28E-013

Figure S8

e

Probe	Symbol	Description	logFC G112 ClQ vs untr	P.Value G112 ClQ vs untr	FDR G112 ClQ vs untr
54873_at	PALMD	palmdelphin	-0,678661644	2,58E-010	3,68E-008
54978_at	C2orf18	chromosome 2 open reading frame 18	0,608072073	4,62E-010	6,03E-008
55062_at	WIPI1	WD repeat domain, phosphoinositide interacting 1	0,937312732	2,51E-015	2,26E-012
55117_at	SLC6A15	solute carrier family 6, member 15	0,995405311	1,69E-015	1,68E-012
55238_at	SLC38A7	solute carrier family 38, member 7	0,588952318	1,38E-009	1,46E-007
55247_at	NEIL3	nei endonuclease VIII-like 3 (E. coli)	-0,676557924	1,34E-010	2,13E-008
55283_at	MCOLN3	mucolipin 3	0,733836575	3,44E-011	6,75E-009
55314_at	TMEM144	transmembrane protein 144	0,923322253	3,17E-013	1,26E-010
55331_at	PHCA	phytoceramidase, alkaline	-0,959334375	2,95E-014	1,89E-011
55740_at	ENAH	enabled homolog (Drosophila)	-0,616169272	6,03E-011	1,04E-008
55902_at	ACSS2	acyl-CoA synthetase short-chain family member 2	0,60113002	1,88E-010	2,89E-008
56180_at	MOSPD1	motile sperm domain containing 1	0,610333374	8,59E-007	3,22E-005
5660_at	PSAP	prosaposin	0,70985959	7,52E-014	3,72E-011
56907_at	SPIRE1	spire homolog 1 (Drosophila)	0,899698274	5,00E-012	1,39E-009
57016_at	AKR1B10	aldo-keto reductase family 1, member B10 (aldose	-0,61216227	0,000299178	0,00407079
57381_at	RHOJ	ras homolog gene family, member J	-0,814788552	2,03E-013	8,38E-011
5803_at	PTPRZ1	protein tyrosine phosphatase, receptor-type, Z poly	-1,005565617	3,42E-014	2,03E-011
58527_at	C6orf115	chromosome 6 open reading frame 115	0,695093156	2,54E-008	1,68E-006
58528_at	RRAGD	Ras-related GTP binding D	1,018370424	4,00E-013	1,55E-010
619190_at	FDPSL2A	MGC44478	0,633450062	0,001407313	0,013270582
6307_at	SC4MOL	sterol-C4-methyl oxidase-like	1,021882824	3,20E-012	9,45E-010
6319_at	SCD	stearoyl-CoA desaturase (delta-9-desaturase)	0,783108932	1,69E-011	3,72E-009
6400_at	SEL1L	sel-1 suppressor of lin-12-like (C. elegans)	0,59677613	2,91E-011	6,00E-009
64121_at	RRAGC	Ras-related GTP binding C	0,913394009	4,49E-015	3,68E-012
643246_at	MAP1LC3B2	microtubule-associated protein 1 light chain 3 beta	0,771138821	3,70E-008	2,30E-006
643428_at	LOC643428	hypothetical LOC643428	-0,837154249	2,37E-009	2,30E-007
6713_at	SQLE	squalene epoxidase	0,600081966	3,55E-011	6,75E-009
677798_at	SNORA9	small nucleolar RNA, H/ACA box 9	-0,629362387	3,73E-007	1,61E-005
6809_at	STX3	syntaxin 3	0,746918644	2,82E-010	3,98E-008
7042_at	TGFB2	transforming growth factor, beta 2	-0,912082592	9,73E-012	2,38E-009

Figure S8

f

Probe	Symbol	Description	logFC G112 ClQ vs untr	P.Value G112 ClQ vs untr	FDR G112 ClQ vs untr
7048_at	TGFBR2	transforming growth factor, beta receptor II (70/80k	-0,585236778	1,34E-008	1,01E-006
7153_at	TOP2A	topoisomerase (DNA) II alpha 170kDa	-0,684794439	5,65E-011	1,01E-008
7316_at	UBC	ubiquitin C	0,671691587	2,02E-007	9,57E-006
7447_at	VSNL1	visinin-like 1	-0,586901243	1,03E-008	8,19E-007
7453_at	WARS	tryptophanyl-tRNA synthetase	0,859124567	4,05E-014	2,23E-011
79993_at	ELOVL7	ELOVL family member 7, elongation of long chain	0,666645437	5,81E-010	7,32E-008
80727_at	TTYH3	tweety homolog 3 (Drosophila)	-0,753878291	1,25E-012	4,04E-010
80818_at	ZNF436	zinc finger protein 436	-0,585473329	3,08E-010	4,27E-008
8165_at	AKAP1	A kinase (PRKA) anchor protein 1	0,708730661	5,92E-011	1,03E-008
81848_at	SPRY4	sprouty homolog 4 (Drosophila)	-0,832261845	1,31E-012	4,19E-010
8404_at	SPARCL1	SPARC-like 1 (hevin)	-0,680409093	4,33E-012	1,25E-009
84418_at	C5orf32	chromosome 5 open reading frame 32	0,669768449	1,55E-011	3,49E-009
84707_at	BEX2	brain expressed X-linked 2	0,628822422	1,65E-008	1,19E-006
8507_at	ENC1	ectodermal-neural cortex (with BTB-like domain)	-0,690003395	9,62E-009	7,71E-007
8553_at	BHLHB2	basic helix-loop-helix domain containing, class B,	0,689247115	3,50E-011	6,75E-009
8878_at	SQSTM1	sequestosome 1	0,788821893	5,42E-013	1,95E-010
891_at	CCNB1	cyclin B1	-0,595714279	1,81E-008	1,28E-006
8934_at	RAB7L1	RAB7, member RAS oncogene family-like 1	0,620423852	5,95E-010	7,45E-008
8974_at	P4HA2	procollagen-proline, 2-oxoglutarate 4-dioxygenase	0,604547489	2,17E-010	3,24E-008
90293_at	KLHL13	kelch-like 13 (Drosophila)	-0,906506831	4,91E-016	6,94E-013
91373_at	UAP1L1	UDP-N-acteylglucosamine pyrophosphorylase 1-lik	0,683352585	9,01E-012	2,26E-009
91445_at	RNF185	ring finger protein 185	0,669045048	1,88E-008	1,33E-006
9156_at	EXO1	exonuclease 1	-0,60152734	7,03E-012	1,85E-009
9388_at	LIPG	lipase, endothelial	0,962640368	4,51E-013	1,69E-010
9493_at	KIF23	kinesin family member 23	-0,599425603	1,11E-009	1,21E-007
9518_at	GDF15	growth differentiation factor 15	0,893113226	1,50E-011	3,41E-009
953_at	ENTPD1	ectonucleoside triphosphate diphosphohydrolase 1	0,690533221	1,08E-010	1,74E-008
96459_at	FNIP1	folliculin interacting protein 1	0,917727705	4,46E-015	3,68E-012
9709_at	HERPUD1	homocysteine-inducible, endoplasmic reticulum st	0,715931664	7,09E-012	1,85E-009
9787_at	DLGAP5	discs, large (Drosophila) homolog-associated prote	-0,627158451	2,95E-010	4,13E-008
9890_at	LPPR4	plasticity related gene 1	0,987214069	7,50E-016	9,28E-013

Figure S8

ClQ_DEGs_#1095 (p53 null)

Probe	Symbol	Descriptio	logFC #1095_ClQ vs untreated	'.Value #1095_ClQ vs un	FDR #1095_ClQ vs untr
10457_at	GPNMB	glycoprotei	3,34	2,10E-16	0,00%
3488_at	IGFBP5	insulin-like	-2,34	2,37E-14	0,00%
2170_at	FABP3	fatty acid b	2,19	1,84E-13	0,00%
8869_at	ST3GAL5	ST3 beta-g	1,92	1,30E-14	0,00%
5046_at	PCSK6	proprotein	1,55	5,60E-12	0,00%
3397_at	ID1	inhibitor of	-1,53	2,31E-10	0,00%
51050_at	PI15	peptidase i	1,44	3,01E-09	0,00%
115265_at	DDIT4L	DNA-dama	1,32	1,33E-10	0,00%
9518_at	GDF15	growth diffe	1,30	1,73E-12	0,00%
79365_at	BHLHE41	basic helix	1,29	7,11E-13	0,00%
1410_at	CRYAB	crystallin, a	1,28	2,27E-12	0,00%
9411_at	ARHGAP2	Rho GTPas	-1,27	7,12E-11	0,00%
5208_at	PFKFB2	6-phospho	1,27	6,31E-11	0,00%
256987_at	SERINC5	serine inco	1,26	2,97E-12	0,00%
344148_at	NCKAP5	NCK-assoc	1,22	1,50E-07	0,00%
7128_at	TNFAIP3	tumor necr	-1,19	1,80E-11	0,00%
23305_at	ACSL6	acyl-CoA s	1,17	1,07E-09	0,00%
64122_at	FN3K	fructosami	1,16	8,78E-09	0,00%
119548_at	PNLIPRP3	pancreatic	1,12	8,40E-08	0,00%
28526_at	TRDC	T cell rece	-1,11	1,12E-08	0,00%
3399_at	ID3	inhibitor of	-1,06	2,02E-10	0,00%
127495_at	LRRC39	leucine ricl	1,03	8,81E-09	0,00%
3675_at	ITGA3	integrin, al	-1,02	5,90E-11	0,00%
9388_at	LIPG	lipase, end	1,01	7,90E-09	0,00%

Figure S9

AAH-APOCIG-1 ARRAY

Each antibody is spotted in duplicate vertically

	A	B	C	D	E	F	G	H
1<br>2	POS	POS	NEG	Akt<br>(P-Ser473)	ATM<br>(P-Ser1981)	BAD<br>(P-Ser112)	Caspase-3<br>(Cleaved Asp175)	Caspase-7<br>(Cleaved Asp198)
3<br>4	CHK1<br>(P-Ser296)	CHK2<br>(P-Thr68)	eIF-2a<br>(P-Ser51)	ERK1<br>(P-T202/Y204)<br>ERK2<br>(P-Y185/Y187)	HSP27<br>(P-Ser82)	IKBa<br>(P-Ser32)	JNK<br>(P-Thr183/Tyr185)	NFKBP65<br>(P-Ser536)
5<br>6	PARP1<br>(Cleaved<br>Asp214/Gly215)	p27<br>(P-Thr198)	P38<br>(P-Thr180/Tyr182)	P53<br>(P-Ser15)	SMAD2<br>(P-Ser245/250/255)	TAK1<br>(P-Ser412)	NEG	POS

Figure S10

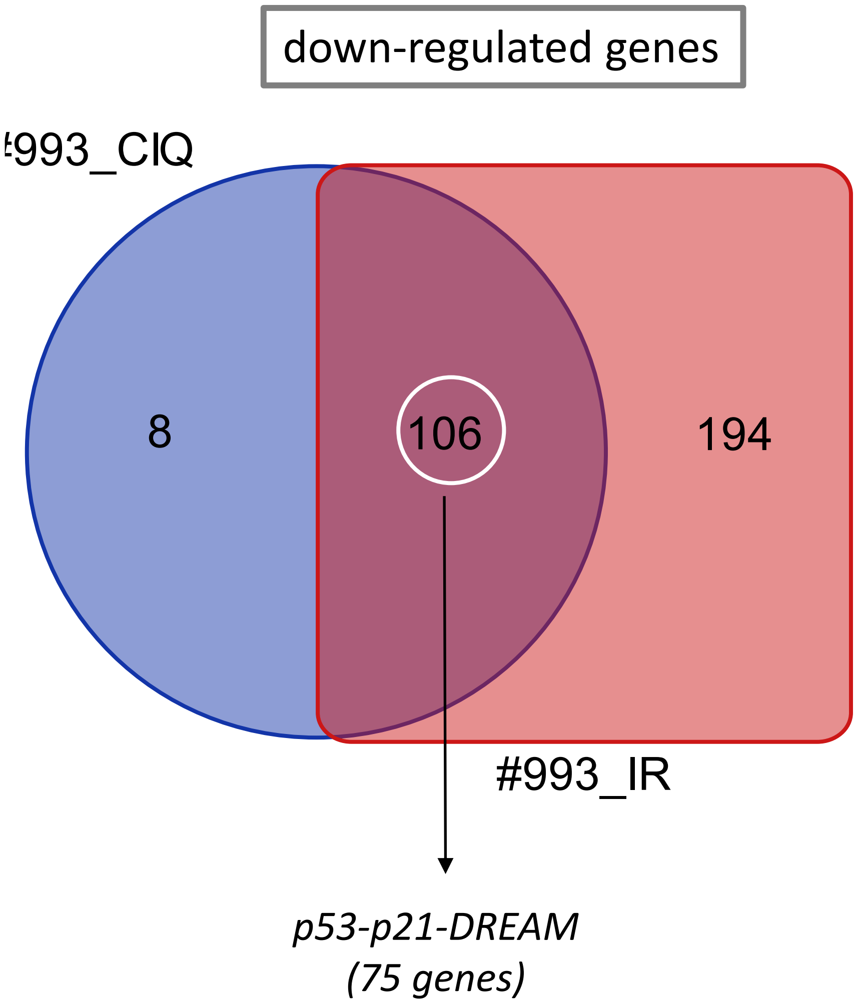
down-regulated genes
993_CIQ
8
106
194
#993_IR
p53-p21-DREAM
(75 genes)

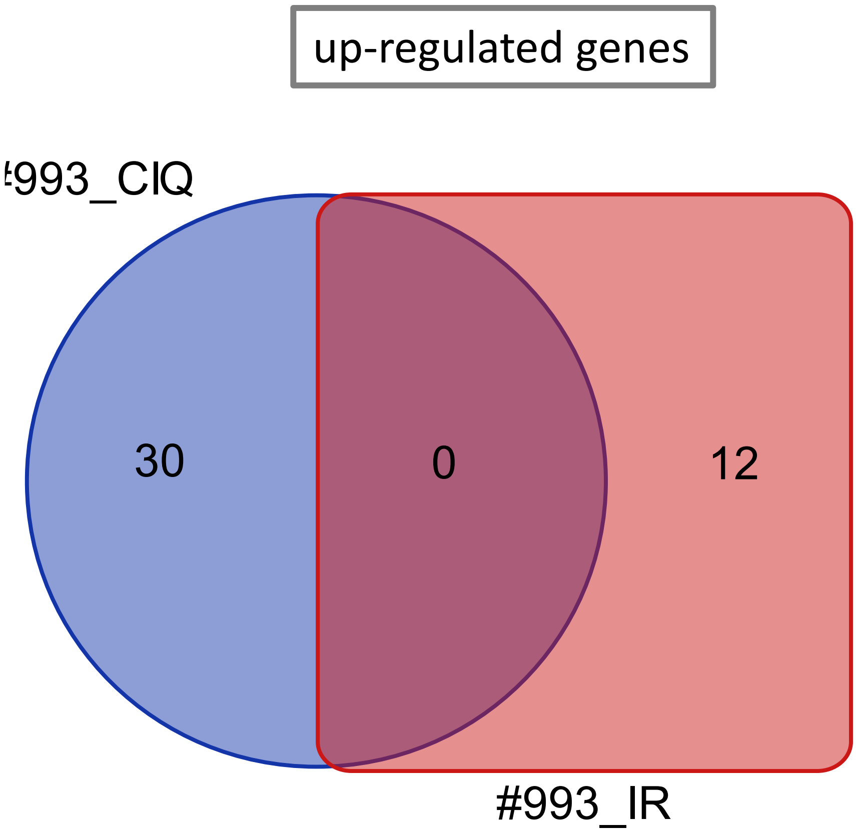
up-regulated genes
993_CIQ
30
0
12
#993_IR

Figure S11

Figure 12 rev

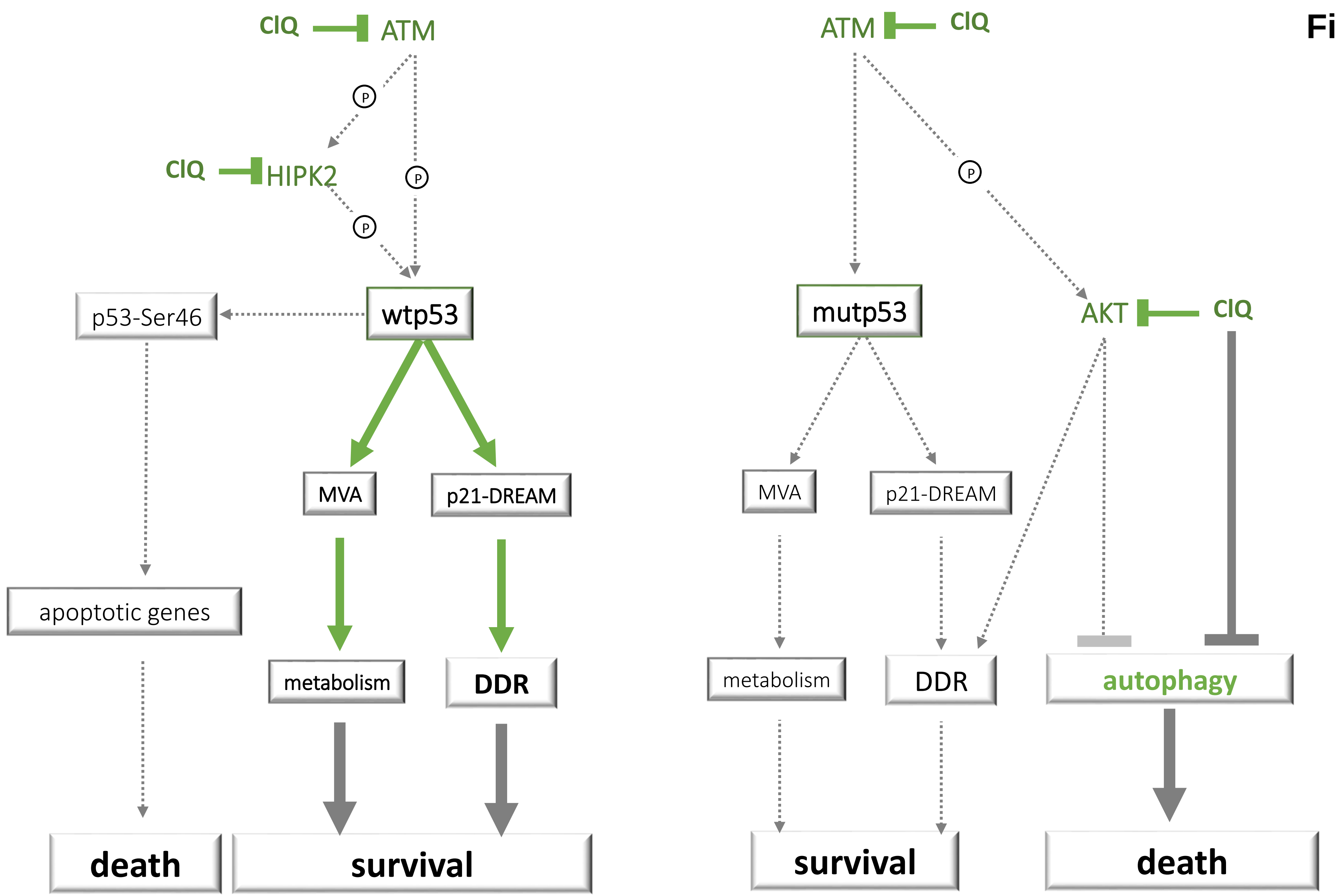

Table S1

a)

**up-regulated ClQ_DEGs**		
**Gene**	**Function in GB**	**Reference**
acsl6	associated with therapy resistance	Zhuo et al. (89)
cryab	associated with therapy resistance	Goplen et al. (90)
ddit4l	associated with therapy resistance	Foltyn et al. (91)
fabp3	associated with stemmness	Lewis et al. (92)
gdf15	associated with poor prognosis	Guo et al. (93)
gpnmb	associated with stemmness	Feng et al. (94)
nckap5	associated with poor prognosis	Akçay et al. (95)
pcsk6	associated with invasion	Delic et al. (96)
pfkfb2	associated with invasion	He et al. (97)
st3gal5	associated with stemmness	Putthisen et al. (98)

b)

**down-regulated ClQ_DEGs**		
**Gene**	**Function in GB**	**Reference**
arhgap29	associated with invasion	Mariani et al. (99)
id1	associated with chemoresistrance	Sachdeva et al. (100)
id3	promotes stemness	Nair et al. (101)
Igfbp5	promotes invasion but inhibits proliferation	Dong et al. (102)
Itga3	promotes radioresistance	Serafim et al. (103)
tnfaip3	promotes GSCs survival and tumorigenicity	Hjelmeland et al. (104)

References

89. Zhuo, S.; Chen, Z.; Yang, Y.; Zhang, J.; Tang, J.; Yang, K. Clinical and Biological Significances of a Ferroptosis-Related Gene Signature in Glioma. Front Oncol 2020, 10, 590861. DOI: 10.3389/fonc.2020.590861
90. Goplen, D.; Bougnaud, S.; Rajcevic, U.; Bøe, S. O.; Skaftnesmo, K. O.; Voges, J.; Enger, P.; Wang, J.; Tysnes, B. B.; Laerum, O. D.; et al. αB-crystallin is elevated in highly infiltrative apoptosis-resistant glioblastoma cells. Am J Pathol 2010, 177 (4), 1618-1628. DOI: 10.2353/ajpath.2010.090063
91. Foltyn, M.; Luger, A.-L.; Lorenz, N. I.; Sauer, B.; Mittelbronn, M.; Harter, P. N.; Steinbach, J. P.; Ronellenfitsch, M. W. The physiological mTOR complex 1 inhibitor DDIT4 mediates therapy resistance in glioblastoma. Br J Cancer 2019, 120 (5), 481-487. DOI: 10.1038/s41416-018-0368-3.
92. Lewis, C. A.; Brault, C.; Peck, B.; Bensaad, K.; Griffiths, B.; Mitter, R.; Chakravarty, P.; East, P.; Dankworth, B.; Alibhai, D.; et al. SREBP maintains lipid biosynthesis and viability of cancer cells under lipid- and oxygen-deprived conditions and defines a gene signature associated with poor survival in glioblastoma multiforme. Oncogene 2015, 34 (40), 5128-5140. DOI: 10.1038/onc.2014.439
93. Guo, L.; Chen, Y.; Hu, S.; Gao, L.; Tang, N.; Liu, R.; Qin, Y.; Ren, C.; Du, S. GDF15 expression in glioma is associated with malignant progression, immune microenvironment, and serves as a prognostic factor. CNS Neurosci Ther 2022, 28 (1), 158-171. DOI: 10.1111/cns.13749
94. Feng, X.; Zhang, L.; Ke, S.; Liu, T.; Hao, L.; Zhao, P.; Tu, W.; Cang, S. High expression of GPNMB indicates an unfavorable prognosis in glioma: Combination of data from the GEO and CGGA databases and validation in tissue microarray. Oncol Lett 2020, 20 (3), 2356-2368. DOI: 10.3892/ol.2020.11787
95. Akçay, S.; Güven, E.; Afzal, M.; Kazmi, I. Non-negative matrix factorization and differential expression analyses identify hub genes linked to progression and prognosis of glioblastoma multiforme. Gene 2022, 824, 146395. DOI: 10.1016/j.gene.2022.146395
96. Delic, S.; Lottmann, N.; Jetschke, K.; Reifenberger, G.; Riemenschneider, M. J. Identification and functional validation of CDH11, PCSK6 and SH3GL3 as novel glioma invasion-associated candidate genes. Neuropathol Appl Neurobiol 2012, 38 (2), 201-212. DOI: 10.1111/j.1365-2990.2011.01207.x
97. He, Z.; You, C.; Zhao, D. Long non-coding RNA UCA1/miR-182/PFKFB2 axis modulates glioblastoma-associated stromal cells-mediated glycolysis and invasion of glioma cells. Biochem Biophys Res Commun 2018, 500 (3), 569-576. DOI: 10.1016/j.bbrc.2018.04.091
98. Putthisen, S.; Silsirivanit, A.; Panawan, O.; Niibori-Nambu, A.; Nishiyama-Ikeda, Y.; Ma-In, P.; Luang, S.; Ohta, K.; Muisuk, K.; Wongkham, S.; et al. Targeting alpha2,3-sialylated glycan in glioma stem-like cells by Maackia amurensis lectin-II: A promising strategy for glioma treatment. Exp Cell Res 2022, 410 (1), 112949. DOI: https://doi.org/10.1016/j.yexcr.2021.112949.
99. Mariani, L.; Beaudry, C.; McDonough, W. S.; Hoelzinger, D. B.; Demuth, T.; Ross, K. R.; Berens, T.; Coons, S. W.; Watts, G.; Trent, J. M.; et al. Glioma Cell Motility is Associated with Reduced Transcription of Proapoptotic and Proliferation Genes: A cDNA Microarray Analysis. J Neurooncol 2001, 53 (2), 161-176. DOI: 10.1023/A:1012253317934.
100. Sachdeva, R.; Wu, M.; Smiljanic, S.; Kaskun, O.; Ghannad-Zadeh, K.; Celebre, A.; Isaev, K.; Morrissy, A. S.; Guan, J.; Tong, J.; et al. ID1 Is Critical for Tumorigenesis and Regulates Chemoresistance in Glioblastoma. Cancer Res 2019, 79 (16), 4057-4071. DOI: 10.1158/0008-5472.can-18-1357
101. Nair, R.; Teo, W. S.; Mittal, V.; Swarbrick, A. ID proteins regulate diverse aspects of cancer progression and provide novel therapeutic opportunities. Mol Ther 2014, 22 (8), 1407-1415. DOI: 10.1038/mt.2014.83
102. Dong, C.; Zhang, J.; Fang, S.; Liu, F. IGFBP5 increases cell invasion and inhibits cell proliferation by EMT and Akt signaling pathway in Glioblastoma multiforme cells. Cell Div 2020, 15, 4. DOI: 10.1186/s13008-020-00061-6
103. Serafim, R. B.; da Silva, P.; Cardoso, C.; Di Cristofaro, L. F. M.; Netto, R. P.; de Almeida, R.; Navegante, G.; Storti, C. B.; de Sousa, J. F.; de Souza, F. C.; et al. Expression Profiling of Glioblastoma Cell Lines Reveals Novel Extracellular Matrix-Receptor Genes Correlated With the Responsiveness of Glioma Patients to Ionizing Radiation. Front Oncol 2021, 11, Original Research. DOI: 10.3389/fonc.2021.668090.
104. Hjelmeland, A. B.; Wu, Q.; Wickman, S.; Eyler, C.; Heddleston, J.; Shi, Q.; Lathia, J. D.; Macswords, J.; Lee, J.; McLendon, R. E.; et al. Targeting A20 decreases glioma stem cell survival and tumor growth. PLoS Biol 2010, 8 (2), e1000319. DOI: 10.1371/journal.pbio.1000319
